# Pediatrics Infected with COVID-19: A Case Series Study on Pediatrics Hospitalized in a Referral Pediatric Hospital

**DOI:** 10.1155/2021/1135503

**Published:** 2021-12-14

**Authors:** Houman Hashemian, Saeid Sadat Mansouri, Hamid Reza Badeli, Ebrahim Esmaili, Majid Asgharzadeh, Tamkin Shahraki, Neda Aligoli Ghasemabadi, Reza Falahatkar, Parham Mashouf, Alireza Jafari

**Affiliations:** ^1^Pediatric Diseases Research Center, 17 Shahrivar Hospital, School of Medicine, Guilan University of Medical Sciences, Rasht, Iran; ^2^Urology Research Center, Razi Hospital, School of Medicine, Guilan University of Medical Sciences, Rasht, Iran; ^3^Inflammatory Lung Disease Research Center, Department of Internal Medicine, Razi Hospital, School of Medicine, Guilan University of Medical Sciences, Rasht, Iran

## Abstract

**Introduction:**

COVID-19 infection which is a novel pneumonia associated with coronavirus suddenly broke out in the world. The aim of this study is to analyze and summarize the clinical characteristics of pediatric patients who were hospitalized in a referral pediatric hospital because of COVID-19 infection.

**Materials and Methods:**

Twenty-one COVID-19 infection cases confirmed by clinical and laboratory findings who were hospitalized in our center from 20 February to 19 April 2020 were included. Demography information, clinical, laboratory, and radiological findings, and treatment strategies of patients were evaluated.

**Results:**

The mean age was 91.5 ± 68.38 months (1-225), and there were 12 (57.1%) boys and 9 (42.9%) girls. Fever ≥ 38°C was detected in 11 (52.4%) patients. Eleven (52.3%) patients had tachypnea, and 4 (19%) of them developed tachycardia. Nine CT scans (42.85%) demonstrated a halo sign, and patchy infiltration was seen in CT scan of 7 patients (33.33%). Furthermore, bilateral crazy-paving pattern was seen in CT scan of nine (42.85%) patients. We prescribed chloroquine in 8 (38.1%), oseltamivir in 8 (38.1%), Kaletra in 6 (28.6%), and Ribavirin in 1 (4.8%) of patients. Finally, four (19.04%) patients expired which one of them suffered from ARDS.

**Conclusions:**

We found out that boys might develop more severe cases of COVID-19, and this could be more common in school age. Manifestations might be milder than adults, and the most severe cases might be associated with underlying diseases. Also, the effectiveness of drugs in the treatment of this disease needs further study.

## 1. Introduction

COVID-19 (novel 2019 coronavirus) infection is currently causing a global pandemic [1]. COVID-19 infection mostly causes respiratory and gastrointestinal symptoms in humans [2], and its clinical manifestations range from a common cold to more severe diseases such as bronchitis, pneumonia, severe acute respiratory distress syndrome, multiorgan failure, and even death [2].

COVID-19 infection has fewer symptoms in children, it is less likely to affect children, it is less severe in them compared to adults [3], and it is also associated with much lower mortality rates in them. The evidence shows that children who suffer from COVID-19 infection are less likely to be symptomatic or develop severe symptoms. Children have gastrointestinal symptoms more often than adults, and most of them develop fever [4].

The majority of children infected by COVID-19 infection mentioned household contact, and neonates who were born from mothers with COVID-19 infection were also in danger [4]. In this prospective study, we wanted to know the characteristics of pediatrics infected with COVID-19 infection in our center, so we collected the demography information, clinical, laboratory, and radiological findings, as well as treatment strategies of our patients.

## 2. Materials and Methods

We investigated the information of pediatrics who were hospitalized in our center, Children's Hospital (Rasht/Iran), from 20 February 2020 to 19 April 2020. Among them, 21 patients were hospitalized because of COVID-19 infection which all of them were confirmed by clinical and laboratory findings, and this was in accordance to the “Diagnosis and Treatment Protocol for COVID-19 (Fifth Revised Edition)” distributed by the National Health Commission [4].

Demography information, clinical, laboratory, and radiological findings, and treatment strategies of pediatrics infected with COVID-19 were gathered in a preprepared form. The demography information consisted of contact history, travel history over the last 2 weeks, hospitalizations history over the last month, influenza vaccine history, routine vaccination history, underlying diseases, using corticosteroid over the last month, chemotherapy or immunosuppressive drugs over the last 3 months, antibiotic consumption over the past two weeks, and types of antibiotics before administration.

In this study by default, the primary chest X-ray was conducted from children admitted to COVID-19 with cough or dyspnea. In the following patients, lung CT (computed tomography scan) was used: in the critically ill patients or in the patient with respiratory distress, in the patients transferred to the intensive care unit, in the patients with bilateral lung involvement based on the chest X-ray, in the patients was getting worse based on the chest X-ray, in the patients with the lack of response to initial treatment, and in the patients with progressive distress. Two experienced pediatric radiologists reviewed them and reported them with results such as ground-glass opacities, consolidations with surrounding halo sign, nodules, fine mesh shadow, pleural effusion, lymphadenopathy, unilateral or bilateral, subpleural/nonsubpleural, and residual fiber strips. Also, we collected the pharyngeal and nasopharyngeal swab samples of the patients to confirm the existence of COVID-19 RNA, and they were verified by a reverse transcription-polymerase chain reaction (RT-PCR).

All the data were entered in IBM SPSS, and all statistical analysis were performed using SPSS version 24.0 software. Total numbers and total percentages of qualitative variables were obtained, and means and standard deviations (SD) of quantitative variables were reported. The formal agreement was taken from every participant in this study, and the protocol for this prospective study was approved by the Ethics Committee of Guilan University of Medical Sciences (IR.GUMS.REC.1399.028).

## 3. Results

### 3.1. Demographic Information

Out of 21 patients with COVID-19 infection, 12 (57.1%) patients were boys, and 9 (42.9%) patients were girls (Table 1). The mean age of pediatric patients with COVID-19 infection was 91.5 ± 68.38 months. The youngest one was 1 month, and the oldest one was 225 months old. 4 (19.4%) pediatric patients expressed contact history with COVID-19 infection cases in their families, and five of them (23.8%) had a history of hospitalizations over the last month. 2 (9.5%) pediatric patients had asthma, and 2 (9.5%) cases suffered from malignancy (Table 1).

### 3.2. Clinical Findings

Regarding complications (Table 2), 11 (52/3%) pediatric patients with COVID-19 infection presented with tachypnea. Among all patients, 4 (19%) of them developed tachycardia. However, two (9%) of them had bradycardia. Also, 2 (9.5%) of pediatric patients with COVID-19 infection suffered from fatigue. Out of 21 pediatric patients, 8 (38.1%) and 4 (19%) patients suffered from dry and wet cough, respectively. None of them complained about sputum during coughing. Four out of 21 (19%) patients complained about diarrhea and nausea during the disease. This study also showed that vomiting existed in 8 (38.1%) pediatric patients with COVID-19 infection (Table 2). Most common symptoms in pediatric patients with COVID-19 infection were fever (52.4%), cough (33.3%), weakness (23.8%), and discomfort in breathing (23.8%) (Table 2). Also, three (14.3%) patients suffered from the acute respiratory distress syndrome (ARDS) and two (9.5%) patients suffered from the acute kidney injury (AKI) (Table 2).

### 3.3. Laboratory Findings

The RT-PCR test was performed on 21 patients, and it was positive in 17 patients (81%) (Table 3). The mean white blood cells (WBC) count was 8460 ± 6438 × 10^9^/L (Table 3), and the mean lymphocytes and polymorph nuclear leukocytes (PMN) count were 28 ± 1963 (×10 9/L) and 64 ± 19.31 (×10^9^/L), respectively. In this study, the mean platelet count of patients was 152290 ± 78030.48 (×10^9^/L), and the mean hemoglobin serum level of patients was 12.33 ± 5.54 (g/L). In addition, the mean value of blood urea nitrogen (BUN) was 24 ± 4796 (mg/dL), and the mean creatinine level of serum was 0.97 ± 0.99 (mg/dL). Moreover, the mean serum electrolyte levels including potassium (K), sodium (Na), and calcium (Ca) were 4.57 ± 1.14 (mmol/L), 131.84 ± 30.53 (mmol/L), and 8.55 ± 1.56 (mg/dL), respectively. This study reveals that coagulation screening tests values were 14.55 ± 1.46 (s) for prothrombin time (PT) and 37.5 ± 9 (s) for partial thromboplastin time (PTT). The findings revealed that the mean international normalized ratio (INR) was 1.26 ± 0.21 in patients with COVID-19 infection. Serum bilirubin levels were 0.9 ± 0.84 (mg/dl) (direct bilirubin) and 1.65 ± 0.77 (*μ*mol/L) (total bilirubin). Alanine aminotransferase (ALT) and aspartate transaminase (AST) were 28.08 ± 21.1 (U/L) and 46.33 ± 24.21 (U/L), respectively (Table 3). The creatine phosphokinase (CPK) was 190.67 ± 162.58 (units/L), and the lactate dehydrogenase (LDH) was 698.71 ± 336.18 (U/L). In this study, a decrease in CPK level was seen in 1 (33%) of patients (Table 3). Also, 3 (42%) of patients had high levels of LDH. Regarding arterial blood gas (ABG), PH of pediatric patients with COVID-19 infection was 7.32 ± 0.11, PaCo2 of patients was 38.91 ± 8.57, PaO2 was 72.80 ± 28.92, and finally, HCO3 of patients was 20.52 ± 5.17 (Table 3).

### 3.4. Radiology Findings

#### 3.4.1. Chest X-Ray

Chest X-ray (CXR) of five pediatric patients with COVID-19 infection (23.81%) out of 21 patients who were included in this study showed peripheral-airspace opacities (Table 4). The study showed that the CXR in five pediatric patients with COVID-19 infection (23.81%) revealed ground-glass opacity characterized by hazy-increased attenuation that did not obscure bronchial and vascular margins of the lung (Table 4). As we showed in Tables 4, 7 (33.33%) pediatric patients with COVID-19 infection had lung consolidations. 6 (28.57%) pediatric patients with COVID-19 infection had some pleural effusions. This study also demonstrated that 4 (19.05%) of the patients had patchy infiltrations. Meanwhile, cavitation was observed in four (19.05%) of them. In addition, 3 (14.28%) of them showed extensive peripheral lymphadenopathy (Table 4).

#### 3.4.2. Lung CT Scans

In this study, four out of 21 pediatric patients with COVID-19 infection received lung CT scans.

The most common findings in lung CT scans were ground-glass opacity and cavitation (66.66%) (Table 4). Whereas, nine CT scans (42.85%) demonstrated a halo sign. More than half of these patients (57.14%) had peripheral-airspace opacities in lung CT scans, and CT scan of ten patients revealed pleural effusion (47.62%). Seven patients' CT scans (33.33%) revealed patchy infiltrations, and bilateral crazy paving pattern was revealed in nine CT scans (42.85%). Eight CT scans (38.09%) showed peripheral lymphadenopathy. Centro lobular nodule was observed in six patients (28.57%), and lung consolidation and reverse halo sign appearance were less common with the incidence of 28.7% and 14.28%, respectively (Table 4).

### 3.5. Treatment Strategies

In this study, nine pediatric patients 9(57.1%) had a history of antibiotics consumption over the past two weeks (Table 5). In the treatment of pediatric patients with COVID-19 infection, chloroquine, oseltamivir, and Kaletra (lopinavir/ritonavir) were used in 8 (38.1%), 8 (38.1%), and 6 (28.6%) of patients, respectively (Table 5). Also, 6 (28.6%), 2 (9.5%), and 2 (9.5%) of patients infected with COVID-19 infection required application of oxygen with mask and oxygen with hood and intubation, respectively. In this study, vancomycin and ceftriaxone were applied to 14.33% of patients with COVID-19 infection during their treatment and ceftriaxone alone in 23.8% of them. Finally, 4 (19.04%) patients died expired, and the other 17 (80.95%) were discharged from the hospital. One of the patients who expired suffered from acute respiratory distress syndrome (ARDS).

## 4. Discussion

COVID-19 viral pneumonia is an acute respiratory infectious disease caused by the novel coronavirus (SARS-CoV-2). The onset of the COVID-19 infection could be asymptomatic (4%) but it often appears as a mild-upper respiratory viral illness (51%) with a lower incidence of pneumonia (39%), and rarely as severe cases such as hypoxia, respiratory distress (5%), ARDS, or multiorgan involvement (<1%) [5]. Since the clinical presentations of pediatric patients with COVID-19 infection are vague or similar to some respiratory infections, it is essential for pediatricians to have more information about COVID-19 infection and its manifestations [6]. In this study, we evaluated the information (demographic, clinical, and para clinical) of hospitalized pediatrics in our center with diagnosis of COVID-19 infection.

In this study, the COVID-19 infection was more common in school-age (Table 1). Also, it was also more common in boys compared to girls which might indicate that boys are more careless about the sanitary protocols at this age, and they might be more likely to be infected with COVID-19 infection. Furthermore, we believe that the age of pediatrics plays an important role on the incidence of COVID-19 infection, and this means that older pediatrics have more social contact and are more likely to being infected with COVID-19 infection. Unlike our results, in the study of Wu et al. [7], 59.5% of COVID-19 infection cases in pediatrics were young girls. However, Hoang et al. [8] revealed that 55.6% of COVID-19 infection cases in pediatrics were boys.

Despite our first assumptions, 12 patients out of 21 had a previous history of contact with COVID-19 cases which means about 50% of pediatric patients with COVID-19 infection had no previous contact history, and this might indicate that other transmission routes are as important as contact history in pediatrics. However, Hoang et al. [8] found that 75.6% of pediatric patients with COVID-19 infection were infected via family members.

Moreover, 8 out of our 21 patients had a previous history of congenital or acquired diseases which might indicate that pediatric patients without underlying diseases are prone to severe cases of COVID-19 infection, as well as pediatrics with underlying diseases such as asthma and malignancies. However, Hoang and colleagues found that immunocompromised pediatrics (30.5%), pediatrics with respiratory (21.0%), or cardiac disease (13.7%) comprised the largest subset of COVID-19 infection pediatrics with underlying disease [8].

This study showed that fever, dyspnea, tachypnea, cough, nausea, and vomiting were the most common and main complaints of pediatric patients with COVID-19 infection, and this was similar to the findings of Wu et al. [7] which demonstrated that the most common symptoms in pediatrics infected with COVID-19 infection were fever (40.5%), dry cough (44.6%), vomiting or diarrhea (21.6%), and headache (3.4%) [7]. Furthermore, Hoang and colleagues [8] showed in their study that the most common symptoms in pediatric patients with COVID-19 infection were fever (59.1%), cough (55.9%), myalgia/fatigue (18.7%), sore throat (18.2%), shortness of breath/dyspnea (11.7%), abdominal pain/diarrhea (6.5%), and rhinorrhea/nasal congestion (20.0%), and 19.3% were asymptomatic.

Currently, there is no certain antiviral drug for the treatment of COVID-19 infection. However, some antiviral and anti-inflammatory agents might have clinically useful impacts on alleviating the manifestations of COVID-19 infection which are induced due to overresponse of immune system through the release of interleukins and other inflammatory agents [9, 10]. For example, some studies have shown that chloroquine could be a potential pharmacologic agent against COVID-19 because of its immunomodulatory effects via inflammatory cytokines inhibition [11], while other studies have shown that it is not effective [12]. However, chloroquine is still considered as a treatment in the treatment guideline of COVID-19 infection in Iran. In this study, 38.1% of pediatric patients with COVID-19 infection were treated with chloroquine, and 28.6% of them were treated with the combination of Kaletra, lopinavir, and ritonavir, and 38.1% of them were treated with oseltamivir. Combination of ritonavir and lopinavir improved patients' symptoms and reduced the need for intensive care unit (ICU) in severe cases similar to the study of Cao et al. [13]. Other hands, many researchers recommended treatments of patients with COVID-19 infection with chloroquine and Kaletra (lopinavir and ritonavir). For example, preliminary results of Huang et al. suggested that chloroquine could be an effective and inexpensive option among many proposed therapies, e.g., lopinavir/ritonavir [14]. However, by passage of times, Jean et al. recommended that remdesivir is the most promising anti-COVID-19 infection agent. In addition, favipiravir and combination therapy with hydroxychloroquine plus azithromycin appear to be acceptable alternatives for the treatment of COVID-19 patients. They believed that for patients with COVID-19 infection, acetaminophen might be a safer agent for treating fever. Finally, low-dose steroid (hydrocortisone) might be prescribed for the treatment of refractory shock in patients with COVID-19 infection [15]. However, it is currently recommended that patients with COVID-19 infection should be better treated with remdesivir with/or without corticosteroids and enoxaparin (Lovenox).

Studies have shown that mortality in children is lower than in adults and varies from about zero to 0.37 percent per thousand people [16]. The mortality is more common in hospitalized children. In a study titled “Demographic predictors of hospitalization and mortality in US children with COVID-19 infection” which was conducted by Moreira et al., in the United States, the death occurred in 0.19% of hospitalized children. Moreira et al. also found that children with a prior medical condition had an increased odd for death [17].

In another study, the mean age of patients who died of definitive COVID-19 infection was 17 years. 86% of patients who died had underlying diseases, especially obesity, asthma, and developmental disorders. The most common complications in death patients with definitive COVID-19 infection were acute respiratory failure and need for mechanical ventilation, acute renal failure, and multisystem inflammatory syndrome in children (MIS-C) [18].

Regarding the radiology and CT findings, Bayramoglu et al. found that the right-sided or bilateral ground-glass opacities were seen in the radiographic findings related to COVID-19 in children. They determined opacities were either single (18.9%) or bilateral (18.9%) around the distal third of the bronchovascular bundle on CT. There was no significant difference in the median size of the largest opacities, total numbers of opacities and involved lobes, and the distance of the closest opacity to the pleura among age groups (*p* > 0.05). The rate of ground-glass opacities with or without consolidation (45.94%) was higher than consolidation alone (16.2%). Feeding vessel sign (43.2%), halo sign (24.3%), pleural thickening (16.2%), interlobular interstitial thickening (13.5%), and lymphadenopathy (8.1%) were other imaging findings [19].

The limitation of our study is the number of cases and the fact that our study focused on more severe cases of COVID-19 infection since our center is a referral hospital. So, this study and its findings should not be generalized to asymptomatic or milder cases of COVID-19 infection. Further well-designed studies with higher number of cases and more precise information and follow-ups are recommended for obtaining more detailed and reliable results.

## 5. Conclusion

We found out that boys might develop more severe cases of COVID-19 infection, and this could be more common in school-age. Manifestations might be milder than adults, and the most severe cases might be associated with underlying diseases. Also, the effectiveness of drugs in the treatment of this disease needs further study.

## Figures and Tables

**Table 1:** Demographic information.

Variable		Mean (SD) or *N*. (%)
Gender	Boy	12 (57.1)
	Girl	9 (42.9)
Age (month)	<1	0
	1-6	3 (14.28)
	6-12	1 (4.76)
	12-72	4 (19.04)
	>72	13 (61.9)
	Mean (SD)	91.5 (68.38)
Weight (g), mean (SD)		26.68 (18.08)
First visit	General physician	10 (47.6)
	Specialist	5 (23.8)
	Hospital	6 (28.6)
Travel history over last 2 weeks	Yes	0
	No	21
History of contact with suspected person	Yes	4 (19.04)
	No	17 (80.95)
History of contact with definite positive person	Yes	3 (14.3)
	No	18 (85.7)
History of hospital visits or hospitalizations over last month	Yes	5 (23.8)
	No	16 (76.2)
History of influenza vaccine	Yes	4 (19)
	No	17 (81)
History of routine vaccination		20 (95.2)
Underlying disease	None	13 (61.9)
	Asthma	2 (9.5)
	Malignancy	2 (9.5)
	Other	4 (19)
History of using corticosteroid over last month	Yes	1 (4.8)
	No	20 (95.2)
Chemotherapy or immunosuppressive drugs over the last 3 months	Yes	2 (9.5)
	No	19 (90.5)
History of consumption antibiotic over past two weeks	Yes	12 (42.9)
	No	9 (57.1)

**Table 2:** Clinical findings.

Variable		Mean (SD) or *N*. (%)
Complains	Fever	13 (61.9%)
	Dry cough	8 (38.1%)
	Wet cough	4 (19%)
	Anorexia	3 (14.3%)
	Diarrhea	4 (19%)
	Nausea	4 (19%)
	Vomit	8 (38.1%)
	Stomachache	3 (14.3%)

Sing and symptoms	Fever	11 (52.4%)
	Chills	1 (4.8%)
	Cough	7 (33.3%)
	Weakness and lethargy	5 (23.8%)
	Discomfort in breathing	5 (23.8%)
	Nausea/vomiting	3 (14.3%)
	Diarrhea	2 (9.5%)
	Retraction sub or intercostal	4 (19%)

Urinary status	Normal	18 (85.7%)
	Oliguria	3 (14.3%)

Pulmonary examination	Fine crackles	3 (14.3%)
	Noise reduction	2 (9.5%)

Temperature in emergency, mean (SD)		37.46 (1.07)
No. heartbeats in emergency, mean (SD)		106.76 (25.9)
No. breaths in emergency (min), mean (SD)		36.48 (15.45)
Tachycardia		4 (19%)
Tachypnea		11 (52.3%)
Bradycardia		2 (9%)

Other findings	ARDS	3 (14.3%)
	AKI	2 (9.5%)

**Table 3:** Laboratory findings.

	Variable	Mean (SD) or *N*. (%)
Blood test	White blood cells (×103/ml)	8460 (6438)
	Lymphocytes (×103/ml)	28 (19.63)
	Polymorphonuclear leukocytes (×10^9^/L)	64 (19.31)
	Hemoglobin (g/L)	12.33 (5.54)
	Platelet count (×10^9^/L)	152290 (78030.48)
	Blood urea nitrogen (mg/dL)	24 (4796)
	Creatinine (mg/dL)	0.97 (0.99)
	Blood potassium level (mmol/L)	4.57 (1.14)
	Blood sodium levels (mmol/L)	131.84 (30.53)
	Blood calcium levels (mg/dL)	8.55 (1.56)
	Prothrombin time (s)	14.55 (1.46)
	Activated partial thromboplastin time (s)	37.5 (9.16)
	Creatine phosphokinase (U/L)	190.67 (162.58)
	Decrease	1 (33)
	Normal	2 (66)
	Lactic acid dehydrogenase (U/L)	698.71 (336.18)
	Increase	3 (42)
	Normal	4 (57)
	Alanine aminotransferase (U/L)	28.08 (21.1)
	Normal range < 45	11 (92)
	>45	1 (8.3)
	Aspartate aminotransferase(IU/L)	46.33 (24.21)
	Normal range < 45	8 (67)
	>45	4 (34)
	Bilirubin direct (mg/dl)	0.9 (0.84)
	Normal range < 0.2	0
	>0.2	2 (100)
	Bilirubin total *μ*mol/L	1.65 (0.77)
	Normal range < 1.2	0
	>1.2	2 (100)

RT-PCR	Positive/negative	17/4 (81/20)

ABG	PH (7.35-7.45)	7.32 (0.11)
	Increase	9 (53)
	Decrease	5 (29.4)
	Normal	2 (18)
	PaCo2 (32-48 mmHg)	38.91 (8.57)
	Increase	3 (17.6)
	Decrease	2 (11.8)
	Normal	12 (70.6)
	Pao2 (83-108 mmHg)	72.80 (28.92)
	Increase	1 (5.9)
	Decrease	8 (47)
	Normal	8 (47)
	HCo3 (20-28 mEq/L)	20.52 (5.17)
	Increase	5 (29.4)
	Decrease	1 (5.9)
	Normal	11 (64.7)
	Base excess	-2.31 (12.01)

**Table 4:** Radiology findings.

Variable	*N*. (%)
CXR	
Peripheral airspace opacities	5 (23.81%)
Ground glass opacity	5 (23.81%)
Lung consolidation	7 (33.33%)
Pleural effusion	6 (28.57%)
Patchy infiltration	4 (19.05%)
Cavitation	4 (19.05%)
Perihilar lymphadenopathy	3 (14.28%)
Lung CT scan	
Peripheral airspace opacities	12 (57.14%)
Ground glass opacity	14 (66.66%)
Lung consolidation	4 (19.05%)
Pleural effusion	10 (47.62%)
Pathchyinfilteration	7 (33.33%)
While lung	6 (28.57%)
Cavitation	14 (66.66%)
Perihilar lymphadenopathy	8 (38.09%)
Crazy paving pattern	9 (42.85%)
Centrolobular nodule	6 (28.57%)
Reverse halo sign	3 (14.28%)
Halo sign	9 (42.85%)

**Table 5:** Treatment strategies.

Variable		*N*. (%)
Drugs except antibiotics	Oseltamivir	8 (38.1)
	Ribavirin	1 (4.8)
	Kaletra: lopinavir/ritonavir	6 (28.6)
	Chloroquine	8 (38.1)

Antibiotics		20 (95.2)

Antibiotic regimen during treatment	Azithromycin	2 (9.5)
	Ceftriaxone	5 (23.8)
	Vancomycin + ceftriaxone	3 (14.33)
	Clindamycin + ceftriaxone	1 (4.8)
	Vancomycin + ceftriaxone + azithromycin	1 (4.8)

Oxygen therapy	Oxygen with mask	6 (28.6)
	Oxygen with hood	2 (9.5)
	Intubation	2 (9.5)
	Oxygen with hood intubation	1 (4.8)

## Data Availability

The data supporting the findings of this study are available from the corresponding author upon request.
